# ENcyclopedia of TRAnscription Factors in Bacteria and Archaea genomes (ENTRAF) version 2.0

**DOI:** 10.1093/database/baaf071

**Published:** 2025-10-29

**Authors:** Silvia Tenorio-Salgado, Cinthia Rodríguez Maya, Edgardo Galan-Vasquez, André Borges Farias, Dulce Álvarez-López, Jose Luis Villalpando-Aguilar, Alberto J Martin, Leonardo Ledesma-Dominguez, Ernesto Perez-Rueda

**Affiliations:** Tecnológico Nacional de México, Instituto Tecnológico de Mérida, Av. Tecnológico km. 4.5, 97118, Merida, Yucatan, México; Centro de Estudios en Computación Avanzada, Coordinación de la Investigación Científica, Universidad Nacional Autónoma de México, Avenida Universidad 3000. Col. UNAM-CU.Coyoacan. 04510, Ciudad de México, México; Instituto de Investigaciones en Matemáticas Aplicadas y en Sistemas, UNAM, Avenida Universidad 3000. Col. UNAM-CU.Coyoacan. 04510, Ciudad de México, México; Laboratório Nacional de Computação Científica—LNCC, Avenida Getúlio Vargas, 25651075, Petrópolis, Rio de Janeiro, Brazil; Instituto de Investigaciones en Matemáticas Aplicadas y en Sistemas, Unidad Académica del Estado de Yucatán, Universidad Nacional Autónoma de México, Carretera Sierra Papacal - Chuburná. Km. 5.5. CP. 97302, Mérida, Yucatán, México; Tecnológico Nacional de México, Instituto Tecnológico de Campeche, Carretera Campeche—Escárcega Km. 9 C.P. 24500 Lerma, Campeche, México; Laboratorio de Redes Biológicas, Centro Científico y Tecnológico de Excelencia Ciencia & Vida, Fundación Ciencia & Vida, Av. del Valle Nte. 725, 8580704 Huechuraba, Región Metropolitana, Chile; Escuela de Ingeniería, Facultad de Ingeniería, Arquitectura y Diseño, Universidad San Sebastián, Bellavista 7, Recoleta, 8580704, Santiago, Chile; Facultad de Ingeniería, División de Ingeniería Eléctrica, Universidad Nacional Autónoma de México, Avenida Universidad 3000. Col. UNAM-CU.Coyoacan. 04510, Ciudad de México, México; Instituto de Investigaciones en Matemáticas Aplicadas y en Sistemas, Unidad Académica del Estado de Yucatán, Universidad Nacional Autónoma de México, Carretera Sierra Papacal - Chuburná. Km. 5.5. CP. 97302, Mérida, Yucatán, México

## Abstract

DNA-binding transcription factors (TFs) have a central role in regulation of gene expression at the transcription initiation level. These proteins have been experimentally described in multiple bacterial and archaeal genomes. These descriptions have allowed their prediction in complete genomes. In this work, we collected 1784 experimentally validated TFs across 25 bacterial and seven archaeal phyla, including Gammaproteobacteria, Bacillota, and Actinomycetota in bacteria and Thermoproteota and Thermococci in archaea. The collection of regulatory proteins was organized into a relational database, named ENcyclopedia of TRAnscription Factors in Bacteria and Archaea genomes or ENTRAF. The database shows the experimental evidence for all the TFs [protein structure information (X-ray or NMR structural data); binding of purified proteins; footprinting assays; site mutation; *in vitro* transcription assay; and PRiMer extension analysis, among others], their global regulatory roles (carbon source assimilation, virulence, antibiotic resistance, stress, and DNA damage), evolutionary families, and structural classifications. In addition, we achieved a global description of the collection in terms of their regulatory mechanisms (activation, repression, and dual activities), structural diversity, functional categories, and protein families. We consider that this collection of well-annotated TFs could be used as a benchmark, enhancing the predictions for this class of proteins in complete genomes. The complete collection of TFs is available at https://entraf.iimas.unam.mx and https://github.com/BioIIMAS/ENTRAF.

## Introduction

How organisms contend against environmental changes depends on their repertoire of genes and their ability to regulate gene expression. It is well documented that gene regulation occurs predominantly at the level of transcription initiation, where the DNA-binding transcription factors (TFs) play a central role [1, 2]. TFs are molecular switches allowing the modulation of the synthesis of specific genes depending on stress responses, metabolic requirements, and food supply, among others [3–5]. This class of proteins specifically binds to DNA-binding sites around or overlapping the promoter, blocking (negative regulation), or allowing (positive regulation) access to RNA polymerase (RNAP), and in consequence, activating or repressing the synthesis of the mRNA [6, 7]. Bacterial activators have been described as proteins that regulate through different mechanisms. For instance, in class I activation, the activator binds upstream and interacts with the carboxy terminal of the α subunit (αCTD) to recruit RNAP to the promoter. In class II activation, the activator binds adjacent to the promoter −35 element and interacts with determinants in RNAP to recruit RNAP to the promoter. On the contrary, a class I activator and a class II activator can work together to recruit RNAP to the promoter [8, 9]. In contrast, repression requires that TFs bind at multiple adjacent sites, with co-operative interactions between repressors bound at adjacent targets, blocking the access of the RNAP to the promoters [10, 11].

Most TFs are mainly two-domain proteins, with a DNA-binding domain (DBD) in either the amino or carboxy terminus that is involved in specific contacts in the DNA, and a ‘Companion domain’ or CD, involved in a plethora of functions such as protein–protein interactions with other proteins (including the transcriptional machinery), ligand-binding (mainly small molecules) [2, 12], and even enzyme activity, or modulating actively the DNA-binding ability of the DBD [13]. In bacteria and archaea, the helix-turn-helix (HTH) domain is the most common DNA-binding motif associated with TFs, followed by nucleic acid-binding domains [14–18].

Given the importance of this class of proteins, their presence and abundance in diverse organisms have been evaluated. From these studies, it has been found that the number of TFs increases from a few hundred in archaea and bacteria, such as *Pyrococcus horikoshii, Bacillus subtilis*, or *Escherichia coli* K12, to over 3000 in *Homo sapiens* [19]. This increment correlates with the hypothesis of genome maturation, where it is proposed that it is necessary a greater number of regulatory elements to regulate a greater number of genes [20]. Consequently, the number of genetic circuits or regulatory networks that arise also increases [21]. Therefore, minor changes in single genes may propagate along such networks and may produce, in the end, quite drastic effects on gene expression in response to external stimuli and changes related to development [22].

On a genomic scale, diverse bacterial organisms have been considered as the archetype to analyse in detail the repertoires of TFs, with *E. coli* K-122, *B. subtilis* 168, and *Pseudomonas aeruginosa*, as the most significant. These organisms have allowed us to explore by sequence comparisons the distribution and abundance of this class of proteins in different organisms [13] and to understand how these proteins are associated with their corresponding regulated genes [23]. From these studies, diverse principles have been elucidated, such as an increase in the number of TFs as a consequence of the genome size [24]; a trend between the number of TFs in relation to the lifestyle, where bacteria with free-living lifestyle also bear a larger number and variety of genes encoding transcriptional proteins than do intracellular pathogens that thrive in more stable biotopes [2, 25]; and that the repertoire of archaeal TFs is similar in sequence to bacterial one, although with different stereochemical mechanisms [13, 26, 27].

In this work, we describe an update of the repertoire of the well-known TFs in Bacteria and Archaea, described as an ENcyclopedia of TRAnscription Factors in Bacteria and Archaea genomes or ENTRAF. To this, TFs were collected from diverse databases and manually curated, increasing the number of proteins devoted to gene regulation, and organized in a website available to the scientific community. In addition, diverse functional characteristics are described, such as the structural diversity of TFs with experimental evidence, and the main functions regulated by these regulatory proteins, among others.

## Material and methods

### Collection of well-known TFs

To obtain an experimentally validated dataset of TFs, we expanded the first version of ENTRAF released in 2020, where an exhaustive search was done. In brief, the SwissProt database (version October 2020) was scrutinized with the following keywords (and their combinations): transcription; gene regulation; DNA-binding; repression; activation; repressor; activator; regulator; bacteria; and archaea. From this search, 668 records were manually filtered and considered as TFs. To do this, we performed literature look-up and BLAST searches against a non-redundant (NR) database at NCBI, with *E*-values less than 10^−3^ to identify proteins with unknown regulatory roles, such as peptidases, integrases, and transporters.

In this version, we describe the main steps to include a regulatory protein in the database, following the strategy previously described in [28]:

(a) A total of 2059 proteins were identified in Swissprot release-2024_01 with the following keywords: transcription gene regulation; DNA-binding; repression; activation; repressor; activator; and regulator, considering the OR conditional. In addition, we used the filters: ‘anti-sigma; sigma’ with the NOT conditional; and constraining the search for ‘bacteria or archaea’ (taxonomy filter). Finally, we selected those reviewed records with the highest annotation score of 5 (reviewed by SwissProt).
(b) A total of 650 proteins were retrieved from specialized and additional databases, such as CollecTF [29], RegulomePA database [30]; RegulonDB [31]; and Protein Data Bank [32]. These proteins were mapped into the initial dataset of TFs.
(c) A total of 275 proteins with significant homology to non-TFs were excluded from the collection. To this end, BLAST searches against an NR database at NCBI, with *E*-values of 10^−3^ and a coverage of at least 70%, were considered. In this step, we identified those proteins with unknown regulatory roles, such as peptidases, lipolytic enzymes, integrases, and transporters. We also searched into the references associated with the protein collection to manually exclude proteins with annotations beyond gene regulation.
(d) A total of 1784 proteins were considered as TFs and included in the collection if they have any of the following 21 experimental categories (included in the database, section Experimental evidences) classified into strong and weak evidences: Assay of Protein Purified to Homogeneity; Binding of Purified Proteins; chromatin immunoprecipitation sequencing (ChIP-seq); Inferred from Direct Assay; mRNA expression levels measured by Quantitative reverse transcription polymerase chain reaction (qRT-PCR); One Hybrid Reporter system; Primer Extension Analysis; Proteomic studies; Protein structural determination; Fur titration assay; Site Mutation; Binding of Cellular Extracts; Gene expression analysis; Author Statement; Inferred from Expression Pattern; Inferred from mutant phenotype; Inferred from Genetic interaction; Reaction Blocked in Mutant; Microarrays; High-throughput transcription initiation; and polyacrylamide gel electrophoresis.
(e) The complete set of sequence proteins included in ENTRAF was analysed by considering InterProScan-5.73-104.0 with the option -pa (pathways), -goterms (GO assignments), -pfam (Pfam-37.2), and supfam (SUPERFAMILY 1.75) (domain assignments).
(f) Finally, to classify functionally the collection of TFs, we followed a similar strategy previously described by Cortes-Avalos et al. [49] associated with the AraC/XylS family. In this regard, an exhaustive association between the TF and its regulated genes was achieved together with the GO annotation directly obtained from protein records and functional annotations of regulatory and binding mechanisms to the gene ontology (GO). For instance, the curator assigned ‘nucleotide metabolism’ to PyrR of *B. subtilis* because this TF regulates transcriptional attenuation of the pyrimidine nucleotide (*pyr*) operon. A similar way was displayed for all the records. Despite this simple classification, some of these proteins are able to regulate the expression of proteins involved in different activities than those previously suggested.

### Web server

**Figure 1.**
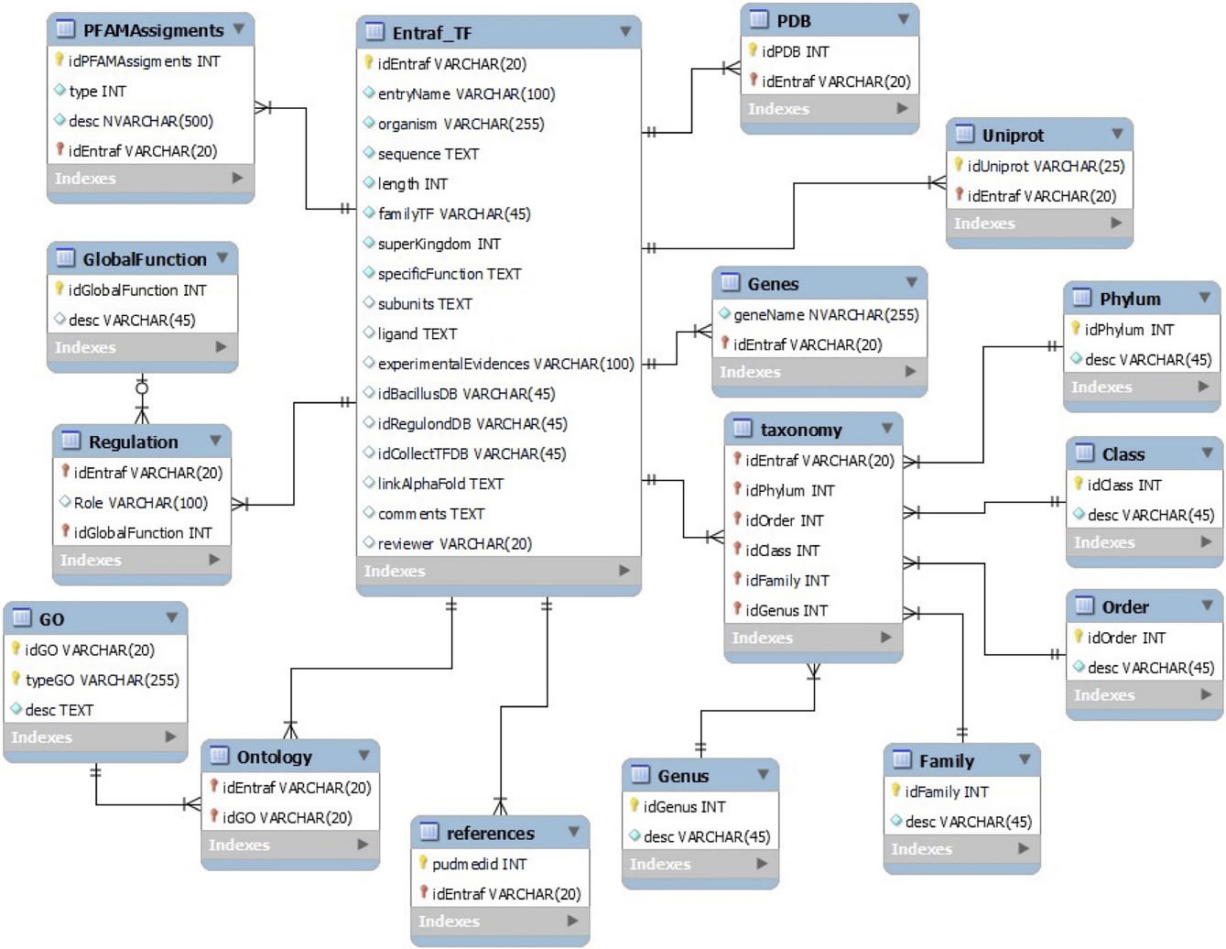
Relational MySQL database. We show the tables and their relations. The whole database in csv format is available at https://github.com/BioIIMAS/ENTRAF/ section data and database (FAQ).

The data of TFs were recorded into a relational MySQL database. The database includes 16 tables, and the master table contains the Entraf_ID, with Uniprot information (entry ID, gene name, and entry name); taxonomy classification (superkingdom, phylum, class, order, family, genus, and organism); protein properties (amino acid sequences and length size), family name; function (specific and global functions), regulatory role (activator, repressor, or dual), GO (biological process, cellular component, molecular function, and IDs), subunits, ligand binding, structural information (PDB, AlphaFold ID), experimental evidences; specialized databases where some records were retrieved (DBTBS, Regulon_DB, and CollectTF), domain organization (SUPFAM and PFAM assignments), comments (functional or structural notes), references (PubMed ID), and reviewer (curator name) (Fig. 1).

In the database, target columns were processed with the full-search text tool to be posteriorly considered in the search in the frontend. The front-end interface has been developed using HTML5 and PHP5 language. It is compatible with Chrome and Firefox web browsers and is designed for mobile, tablet, and desktop. The information can be accessed by protein ID, family name, or organism name, among others, making queries to it. Finally, all information can be downloaded from a raw file included on the webpage. The complete database can be downloaded from the ‘FAQ’ section, ‘How can I get the complete database?’

## Results

**Figure 2.**
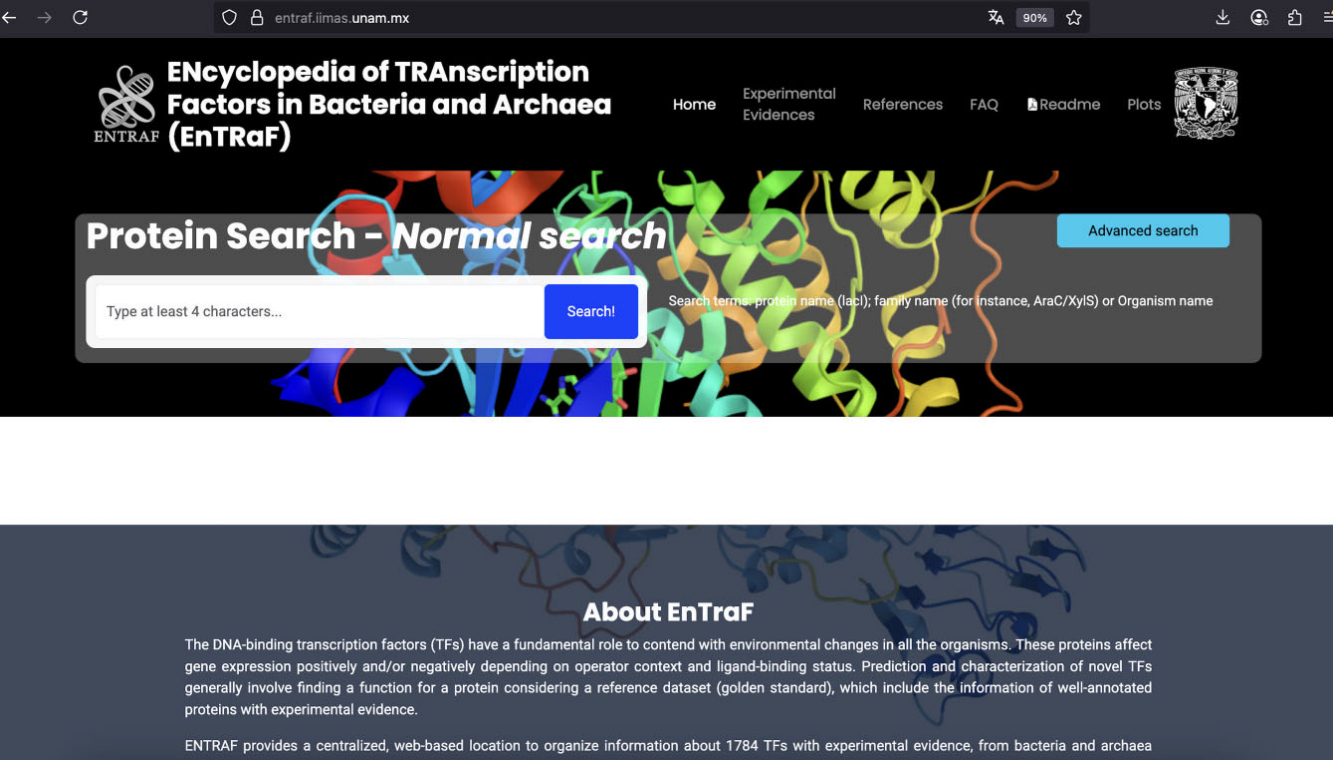
Website of ENTRAF. Information of 1784 transcription factors with experimental information was included in the database.

In this work, we described the ENTRAF database, which contains an extended collection of TFs in bacteria and archaeal organisms (Fig. 2). In contrast with organismal-specific databases, such as RegulonDB (*E. coli* K-12) or DBTBS (*B. subtilis* 168) or databases with DNA-binding sites (CollecTF), ENTRAF is specialized into proteins classified as DNA-binding TFs and considers information of 411 different bacterial and archaeal organisms, associated with 25 phyla, increasing the coverage of TFs in bacteria and archaea, in relation to the 15 phyla included in CollecTF. In this regard, ENTRAF also integrates the organismal information from the databases previously described, such as those 212 TFs from RegulonDB, 120 from DBTBS, and 318 from CollecTF. However, this information represents the 36% of the complete collection described in this work. In addition, ENTRAF expands the experimental evidences associated with TFs and classifies them into 28 categories, such as proteins with structural data, mutations, or footprinting information, among others.

### Database

**Figure 3.**
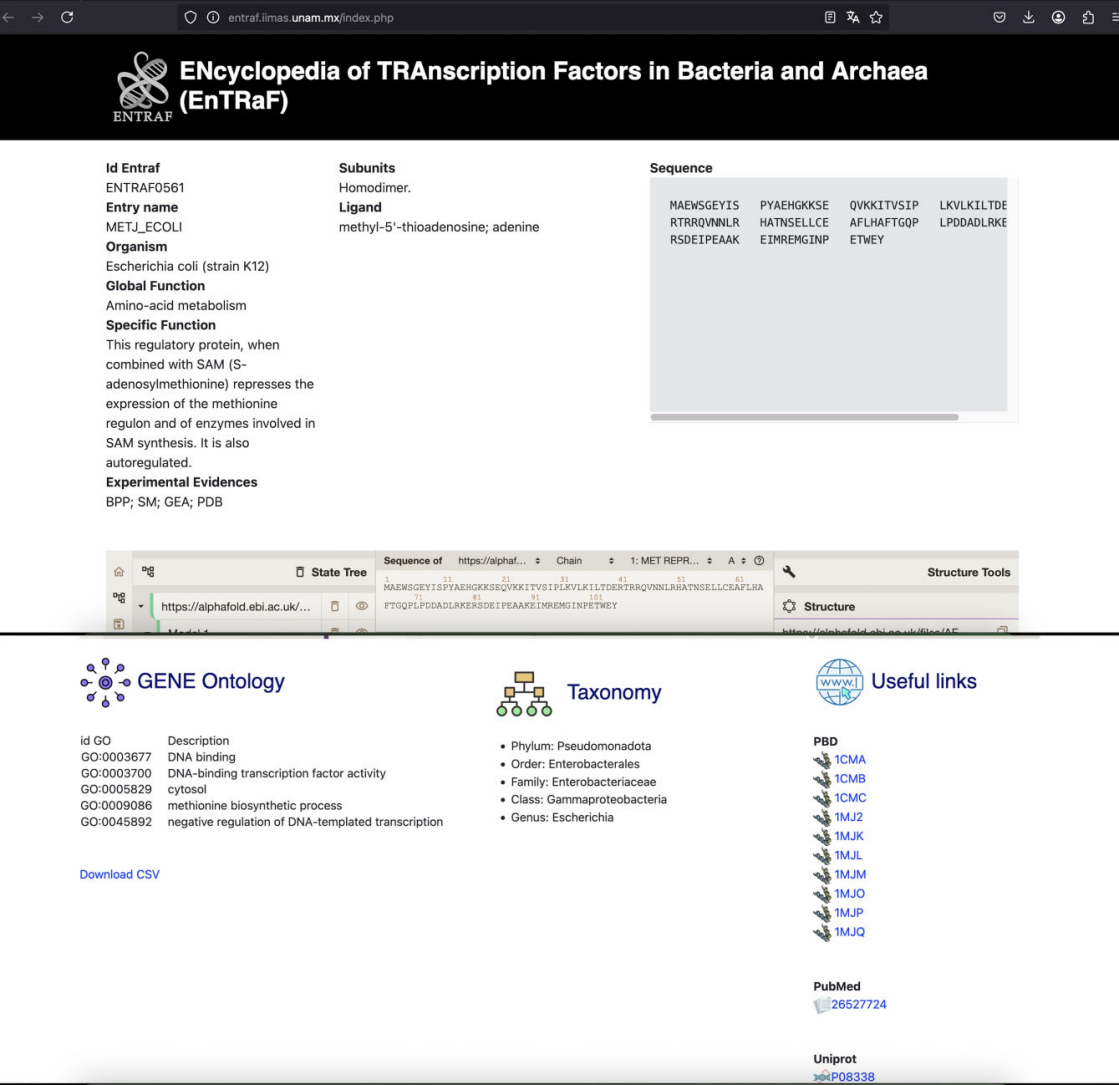
Information of MetJ in the bacterium *E. coli* is displayed. The EnTRaF ID, entry name, organism, global and specific function, subunits, ligands, sequences, AlphaFold model, gene ontology, taxonomy, and links to PBD, PubMed, and Uniprot are displayed.

Figures 2 and 3 show the ENTRAF website, where the collection can be accessed by basic and advanced searches. In basic searches, diverse terms can be used, such as the family name (AraC/XylS), the TF name (MetJ), or by organism (*E. coli*). In advanced search option, users can search through the search fields: GO, functional category, and taxonomy. The resultant page displays a table with the TFs with their functional characteristics, deposited in the database. A README file is also included in the database.

### Taxonomic distribution

**Figure 4.**
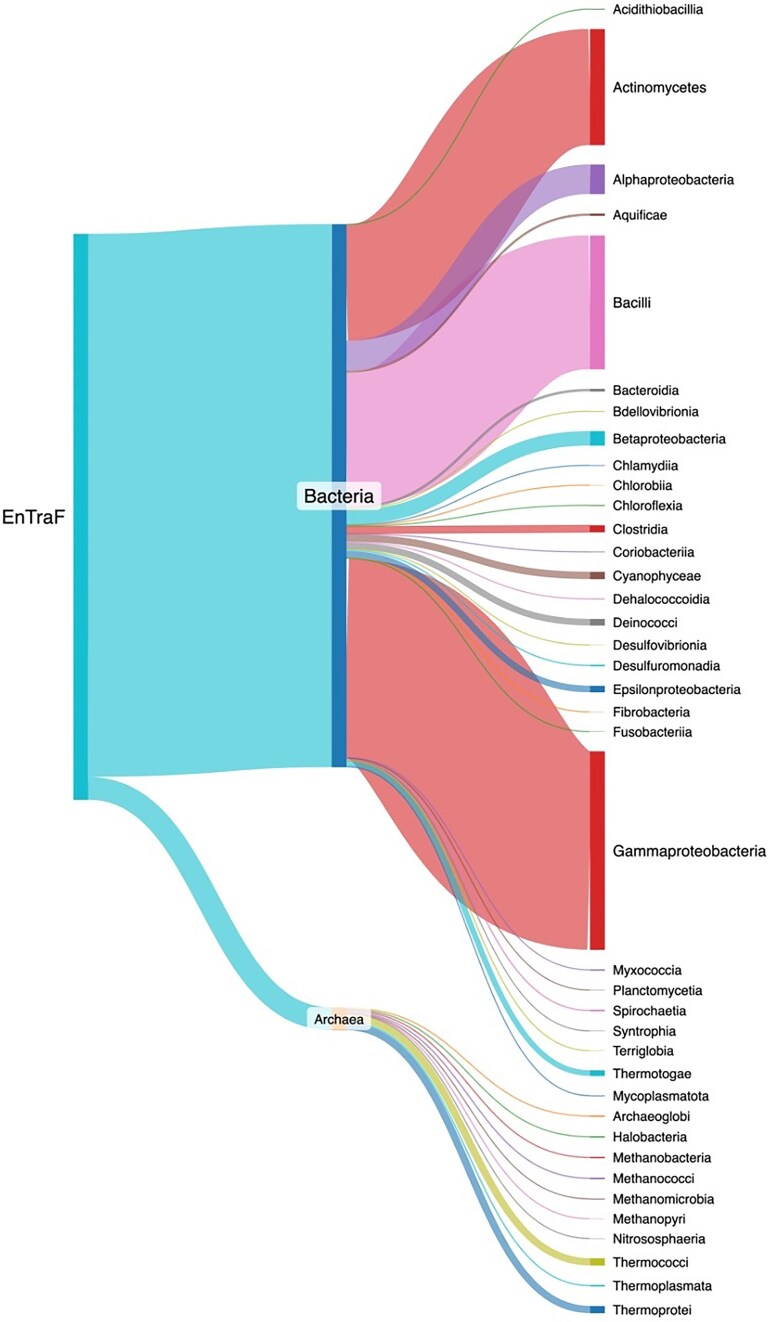
Taxonomic distribution of the TFs included in ENTRAF. Bacterial TFs were identified in 29 orders, whereas archaeal TFs have been characterized into 10 orders. See text for details. A dynamic figure describing the taxonomic distribution for all the TFs has been included as Supplementary Material S1, https://github.com/BioIIMAS/ENTRAF/, and database (Plots).

The ENTRAF version 2.0 contains all the following information: protein name, organism, taxonomic lineage, global function, regulatory role, ligand, and structural information. The literature curation is up-to-date within 24 months and includes 2787 references. In this version, a total of 1784 TFs were identified and deposited in the collection, i.e. 1116 new records in relation to the first release [28]. From these, 1712 TFs have been characterized in 29 bacterial orders and 72 TFs in 10 archaeal orders (Fig. 4 and Supplementary Material S1). 36.5% of the bacterial TFs have been experimentally described in Gammaproteobacteria, followed by Bacilli with 24.6% of the bacterial TFs and Actinomycetota with 21.3%. These three phyla represent a total of 82.5% of the bacterial collection. The other 17.4% is associated with 26 different orders. Concerning the 72 archaeal TFs, they have been mainly characterized in Thermoprotei and Thermococci, the orders with the most TFs described so far, 23 each, representing 63.8%. The other 36.2% is associated with eight different orders.

### Structural classification of transcription factors

**Figure 5.**
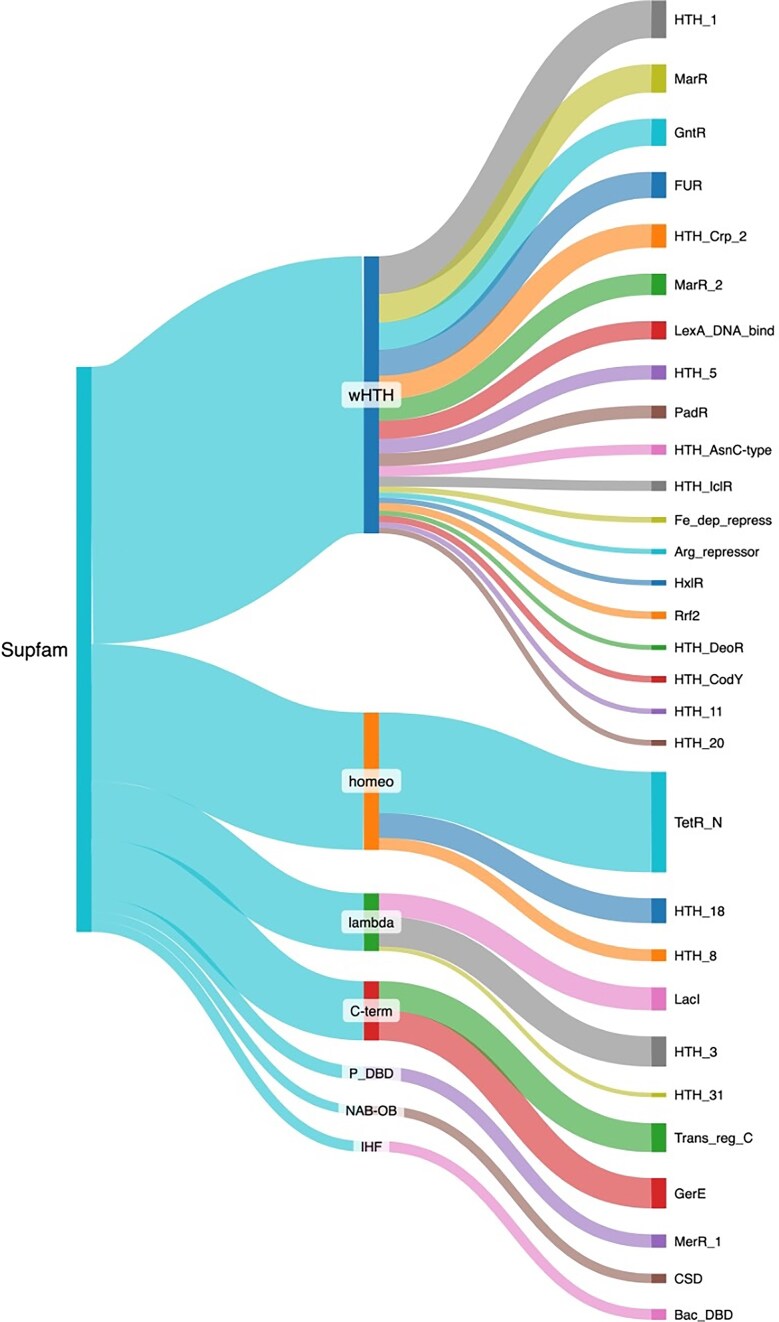
Structural classification of the TFs included in ENTRAF. Superfamilies with more than 10 proteins were displayed, such as wHTH, homeo, lambda, C-Term, P-DBD, NAB-OB, and IHF. In addition, families determined by PFAM are also displayed. See text for details. In Supplementary Material S2, https://github.com/BioIIMAS/ENTRAF/, and database (Plots), all the superfamily and family assignments are provided.

Structural analyses have evidenced the existence of DBD structural families. These superfamilies have been defined by considering the conservative definition in the SCOP database [33]. Therefore, to have a perspective of the structural diversity associated with the collection of TFs, the repertoire of proteins deposited in ENTRAF was classified into 18 superfamilies, according to the superfamily database [34] (Fig. 5 and Supplementary Material S2). The most abundant is the winged helix DNA-binding domain (wHTH), with 623 members, followed by the homeodomain-like (Homeo) with 309 members, the C-terminal effector domain of the bipartite response regulators (C-term) with 133 proteins, and lambda repressor-like DBDs with 130 members. In minor proportions were identified 14 superfamilies, such as the ribbon–helix–helix, TrpR-like, IHF, and AlbA-like. In the following, we describe some superfamilies, and their families identified in the collection of ENTRAF.

The wHTH motif is an extension of the HTH group, which is characterized by the presence of a third ɑ-helix and an adjacent ß sheet, which are components of the DNA-binding motif. The recognition helix binds as in the regular HTH motifs, and the extra secondary structural elements provide additional contacts with the DNA backbone [35, 36]. In ENTRAF, the wHTH represents the superfamily with the largest number of families, with 33, where members of the Crp (PF13545), AsnC (PF13412), and LysR (PF00126) families are included. In this regard, the catabolite gene activator (CAP) is a cAMP-dependent transcription regulator. A rise in cAMP concentration leads to increased affinity of CAP for catabolite-sensitive operons. The protein functions as a homodimer, and each subunit comprises a two-domain structure. The carboxy-terminal domain (about 60 residues) mainly consists of a three-helix bundle, with the second two helices forming the HTH motif. The domain contains a small ß sheet that also contributes to DNA binding. The larger amino-terminal domain (~130 residues) has an extensive ß sheet that mediates cAMP binding, and a long helix that forms the dimer interface [37, 38]. Binding by the recognition helix of the HTH motif in the major groove induces a sharp, highly localized bend in the DNA, and additional contacts with the phosphate backbone are made by the ß strands from the same domain.

The C-term superfamily includes the transcriptional regulatory protein, C-terminal. The structure of the C-term DBD consists of three α-helices packed against two antiparallel β-sheets, an N-terminal four-stranded antiparallel β-sheet and a C-terminal hairpin. The hairpin interacts with a short stretch of β-strand that connects helices α1 and α2 to generate a three-stranded antiparallel β-sheet. The hydrophobic core of the domain is formed by sidechains contributed by each of the seven β-strands and three α-helices [34, 36, 39–42]. In this group, 133 response regulators associated with the OmpR (PF00486) and LuxR (PF00196) families are included.

Proteins associated with the lambda repressors exhibit two domains: an amino-terminal five-helix bundle whose second and third helices comprise an HTH motif; and a carboxy-terminal domain that mediates dimerization. The recognition helix of the HTH motif contacts base edges in the DNA major groove [43]. For instance, the purine repressor proteins of the LacI repressor family regulate *de novo* purine and pyrimidine synthesis by repression of genes encoding enzymes that participate in the synthesis pathway. Guanine and hypoxanthine act as co-repressors on binding to the protein. Other members of the LacI repressor family display high structural and sequence similarity and control a wide range of biosynthetic pathways [43–46]. The amino-terminal domain of PurR (~60 residues) contains a three-helix bundle followed by a loop and an additional helix. The first two helices form the HTH motif, and the fourth is called the hinge helix. The larger carboxy-terminal domain (about 280 residues) is a mixture of helices and ß strands and binds the co-repressor. Binding sites are typically 16–18 bp long and pseudo-palindromic. The recognition helix of the HTH motif binds in the major groove, and phosphate backbone contacts are mediated by the remainder of the helical bundle. The hinge helix from each subunit is inserted in the same DNA minor groove at the center of the binding site and jointly introduces a kink by intercalation of leucine sidechains [15, 35, 47, 48]. In ENTRAF, eleven families were identified as associated with this superfamily, such as LacI (PF00356) and Cro/C1 (PF13443), among others.

Transcription regulators with homeodomains are small (just over 100 amino acid residues in length) and consist of four helices. The protein binds DNA either as a monomer or a dimer, depending on the protein, and many are capable of both. Typically, the second helix of the motif is inserted in the DNA major groove. In the collection, 339 proteins associated with the Homeodomain-like family were identified. The TetR/AcrR (PF00440) family, which includes 68% of the homeodomain proteins. Seven additional families constitute the other 32% of these proteins, such as the AraC/XylS (PF12833) and Fis (PF02954) [49, 50].

The members of the ribbon–helix–helix group use smaller two- or three-stranded ß sheets or hairpin motifs to bind in either the DNA major or minor grooves. Six protein families were identified in the collection, represented by the MetJ repressor and the Arc repressor. The MetJ and Arc repressors are both dimers with very similar modes of binding. Each protein subunit comprises a helical bundle and a single ß strand; the strands from each subunit pack side by side, forming an antiparallel sheet that binds in the DNA major groove. The sheets lie flat in the groove; therefore, protein side chains from just one face of the strand interact with base edges [51–55]. In this superfamily, 16 protein families were included, associated with the MetJ (PF01340), Rhh (PF01402), Arc (PF03869), Omega repressor (PF07764), DBD of proline dehydrogenase (PF14850), and CopG (PF16777).

### General functions associated with transcription factors

TFs from diverse families, such as AraC/XylS and LysR, have been classified by the genes they regulate, such as those involved in (1) the regulation of carbon metabolism, (2) stress response, or (3) virulence [49, 56]. In this regard, we manually curated and extended the classification to the whole collection of 1784 TFs included in ENTRAF. Therefore, we propose 30 classes or groups, including one non-determined function. In general, stress response (TFs associated with response to pH changes, heat-shock response, DNA damage, cold-shock response, and starvation response, among others); carbon source metabolism (regulators involved in lactose, maltose and maltodextrin, or catabolite repression, among other carbon sources assimilation); virulence (TFs devoted to regulating genes of virulence factors, such as toxin regulation, alpha-hemolysin regulation, extracellular polysaccharide synthesis, and flagellum synthesis, among others), and antibiotic response (expression of beta-lactamase, erythromycin, tetracycline, and lincomycin-resistance, among others). With this classification in mind, we found that 36% of the total collection are associated with stress responses, carbon sources metabolism, virulence, and antibiotic resistance. It is important to emphasize that this classification is useful to determine the general role of the TFs; however, some of them regulate the expression of genes involved in different activities than those previously suggested. Additionally, it is important to mention that metabolism genes are also involved in the virulence process and that the encounter of a pathogen with the immune system involves stress. For instance, PtxR (PTXR_PSEAE) regulates factor virulence exotoxin A genes in *P. aeruginosa*, and it has been associated with the positive regulation of glucose metabolism via the regulation of the expression of the *kgu* and *gad* operons [57–59]. Therefore, in the following, we describe the most relevant functional categories of ENTRAF.

### Stress responses

Proteins classified as stress response regulators constitute 13.3% of the ENTRAF collection, and include TFs associated with redox responses, temperature responses, or oxidative stress. For instance, MprA of *Mycobacterium tuberculosis* (MPRA_MYCTU) belongs to the MprB/MprA system that is involved in the regulation of numerous stress-responsive genes, including up-regulation of two sigma factors, *sigE* and *sigB*, as well as *pepD* and *mprA*, and repression of multiple genes from regulons associated with hypoxia, starvation, and iron metabolism [60, 61]. The majority of genes regulated by MprB/MprA under a particular stress condition are different from those induced during normal growth, but several genes are commonly regulated under more than one condition [61, 62]. Another protein associated with stress responses is Anr of *P. protegens* (ANR_PSEFL), a transcriptional activator involved in the positive regulation of the components of the hydrogen cyanide synthase (*hcnABC*) [63–65]; or members of the family cold shock proteins (CSPA_BACCE, CSPA_BACSU, among others) involved in the cell response at low temperatures [66, 67]. Finally, the redox- and pH-responsive transcriptional regulator WhiB3 of *M. tuberculosis* (WHIB3_MYCTU) has been involved in the maintenance of intracellular redox homeostasis by regulating catabolic metabolism and polyketide biosynthesis. Regulates expression of the redox buffer ergothioneine (ERG) in a carbon-source-dependent manner, and in response to low external pH alters endogenous gene expression, leading to acid resistance [68, 69].

### Carbon sources assimilation

In ENTRAF, 9.5% of the TFs are associated with the regulation of carbon source metabolism, where those TFs involved in catabolite repression, xylose, or maltose assimilation stand out. In this regard, the collection includes the classical lactose repressor of *E. coli* (LACI_ECOLI) or DasR of *Streptomyces coelicolor* (DASR_STRGR) associated with the repression of the phosphotransferase system (PTS) specific for the uptake of *N*-acetylglucosamine (PTSNag), and genes involved in the metabolism of chitin, as well as several genes involved in development, thereby linking carbon availability to morphogenesis [43, 70–72].

### Virulence

Proteins involved in the regulation of virulence genes correspond to the 6.8% (i.e. 121 TFs) of the total TFs in ENTRAF. Those proteins have been mainly experimentally characterized in 25 different bacterial species, where it stands out *Mycoplasma tuberculosis* with 34 TFs, and *Staphylococcus aureus* with 45 TFs. In general, these TFs are involved in the regulation of secretion systems, stimulation of anthrax toxin expression, hemagglutinin/protease regulatory protein, and virulence factors alpha- and beta-hemolysin, such as AgrA that regulates the quorum-sensing response in *S. aureus* (AGRA_STAAU) and controls the production of hemolysins and other virulence factors [73, 74]. AgrA binds to DNA via its C-terminal LytTR domain, a domain not found in humans but common in many pathogenic bacteria, making it a potential target for antimicrobial development [75]. Another interesting example corresponds to VirS of *M. tuberculosis, a member of the* AraC/XylS family, which is induced in low pH, is required for the intracellular pH maintenance in response to acidic stress and inside acidified macrophages, playing a central role in blocking phagosomal-lysosomal fusions [76]. Transcriptomics experiments revealed that VirS affects the expression of genes encoding metabolic enzymes, cell-wall envelope proteins, efflux pumps, ion transporters, detoxification enzymes, and transcriptional regulators expressed under low-pH stress [76, 77]. Therefore, the TFs, included in this functional category, have gained notoriety as a target for multiple drugs.

### Antibiotic response

TFs involved in antibiotic resistance correspond to 6.3% of the total of regulatory proteins deposited in ENTRAF. In this collection, members of the TetR/AcrR family are mainly associated with the sensing and responding to various structurally dissimilar antimicrobial agents. Upon detecting these agents, the regulators allow transcription of an appropriate array of resistance markers to counteract the deleterious compounds. *Campylobacter jejuni* CmeR (Q0PBE2_CAMJE) is a pleiotropic regulator of multiple proteins, including the membrane-bound multidrug efflux transporter CmeABC [78–80]. CmeR represses the expression of *cmeABC*, and it is induced by bile acids, which are substrates of the CmeABC tripartite pump. The multiligand-binding pocket of CmeR has been shown to be very extensive and consists of several positively charged and multiple aromatic amino acids [78, 81].

### Functional processes are mainly negatively regulated

To understand the regulatory role of TFs in ENTRAF, we determined their main regulatory processes associated with these proteins. In general, most of the TFs remain with an unknown function, probably because they are not totally characterized and are products of global experiments, such as ChIP-seq or structure characterizations. Indeed, almost 54% of the TFs included in the dataset remains to be determined if they are repressor and/or activators. Concerning the rest of the proteins in the collection, we found that 824 TFs have mainly been identified as repressors with 22.12%, followed by dual activity (12.45%) and activators (11.45%). This finding is consistent with the description of regulation in the bacterium *E. coli* K12, where a trend of repression mechanisms was observed [31].

**Figure 6.**
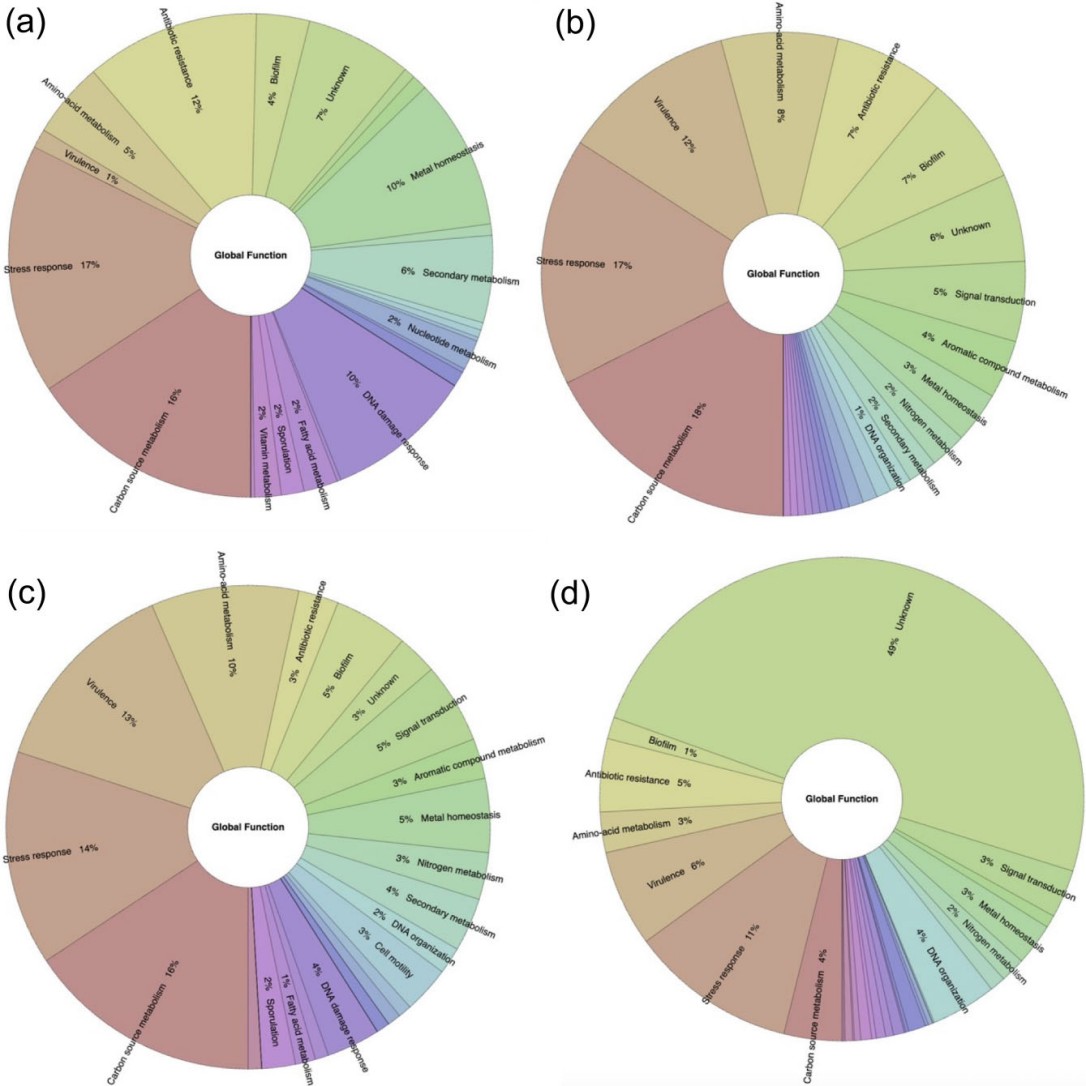
Functional groups are preferentially (a) activated, (b) repressed (red), (c) dual, and (d) unknown regulated by TFs included in ENTRAF (https://github.com/BioIIMAS/ENTRAF/) and database (Plots). See text for details.

We also asked if the global functions are mainly repressed or activated. In this regard, we identified that repression is the main mechanism associated with eight functional categories (stress response, carbon source metabolism, antibiotic resistance, metal homeostasis, DNA damage response, secondary metabolism, nucleotide metabolism, fatty acid metabolism, and vitamin metabolism), with an average of 46.7% of the TFs described as repressors. In two functional categories (biofilm and aromatic compound metabolism), the activation is predominant, with an average of 29.2% of their TFs associated with positive regulation. Concerning the dual activities, they are mainly associated with sporulation, cell motility, signal transduction, virulence, amino acid metabolism, and nitrogen metabolism. Finally, in two categories (cell division and DNA organization), unknown functions are predominant (Fig. 6 and Supplementary Material S3).

In summary, 30 global functions included almost all the TFs identified in the collection; however, they must be considered as a first step to understand globally the collection of regulatory proteins. Indeed, their specific role in diverse bacteria and archaea depends on their network interaction for controlling gene expression.

### TF families regulate similar functions

**Figure 7.**
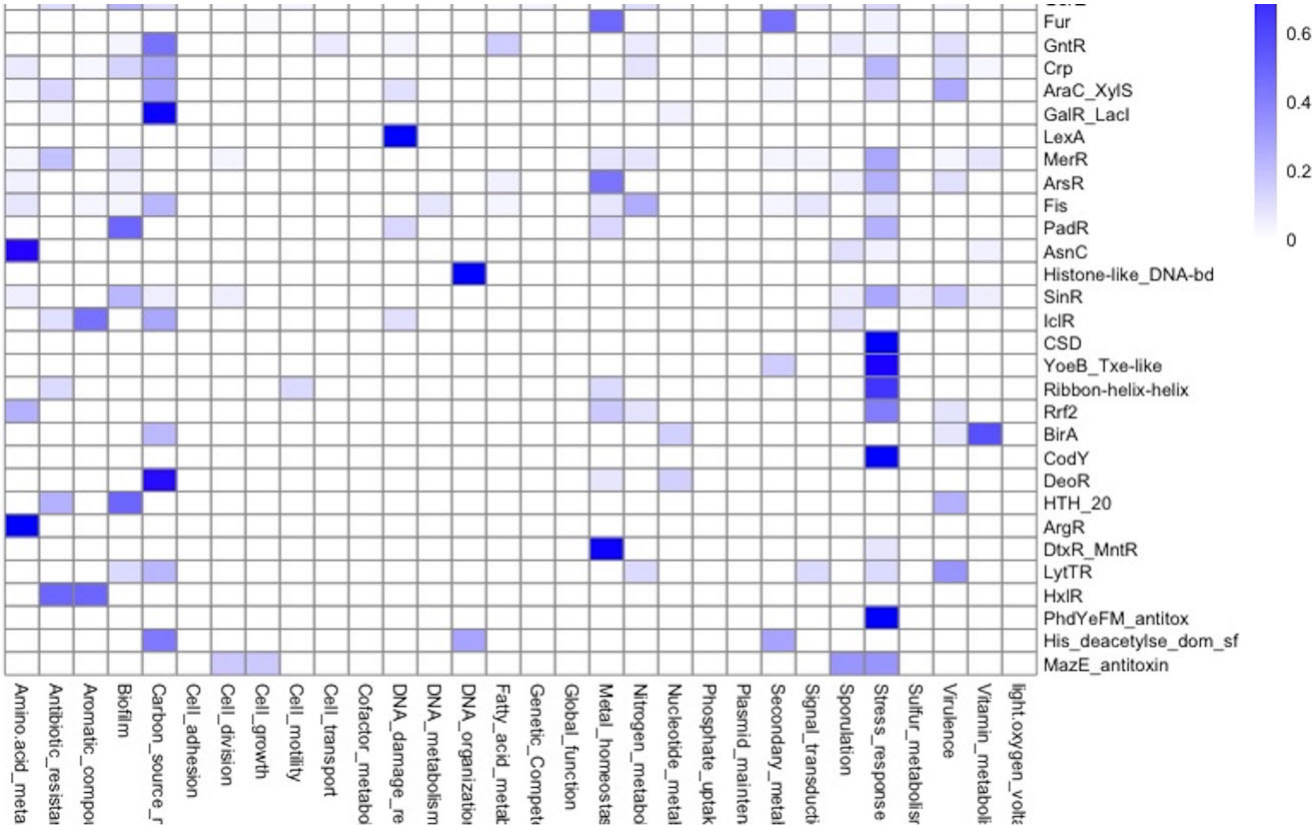
Functional groups in association with protein families. Families with more than 10 members were included in the plot. The bar indicates the proportion of families by functional category (0.0 low and 1.0 maximum association).

A natural question to explore is the relationship between global function and evolutionary families, where TFs have been classified. In this regard, the distribution of functional roles suggests that families such as TetR/AcrR proteins are mainly devoted to regulating antibiotic-resistance genes; ArgR, AsnC, and LysR are involved in the regulation of amino acid metabolism; the families GalR/LacI, GntR, and DeoR are associated with carbon sources metabolism; LexA with DNA damage response, and histone-like DNA-binding proteins are involved in DNA organization or cold shock domain proteins associated with stress responses; and fur regulators associated with ‘metal homeostasis’ and ‘secondary metabolism’ (Fig. 7). This functional relation between regulated functions and families suggests conservation of the regulatory roles of its members. In this regard, members of the Trans_reg_C family (where OmpR and PhoB regulators are included) are mainly involved in the regulation of genes related to the biosynthesis of membrane components in bacteria accordingly, were associated with the categories ‘signal transductions, stress response and virulence’. These observations lead us to conclude that the regulatory mechanisms of TF families may be conserved across genomes with some organism-specific or lineage-specific variations, as it has been previously described for *E. coli* and *B. subtilis* [82].

### Future directions

Since ENTRAF is an interactive database, one of its updates will come from the academic community interested in gene regulation and in particular in TFs; therefore, we will present a summary of the updates once a year. In addition, we consider that the use of artificial intelligence tools will improve how we gather the evidence and incorporate it into the curation processes. In this regard, approaches such as natural language processing strategies will allow to fill the information in future updates. We also consider that the use of gene expression and co-expression analysis will help to determine the specific role of each TF. Finally, we consider that the expansion of the collection with AI-based predictions will also be included in the future versions of ENTRAF.

## Conclusions

ENTRAF represents a comprehensive collection of TFs experimentally characterized in multiple bacterial and archaeal organisms that could be used for comparative genomics analysis. The collection includes structural and functional information, such as families, superfamilies, ligand-compounds, regulatory roles, global regulatory functions, experimental evidence, and taxonomical distributions, among others. To reinforce the experimental evidence, 2778 references were considered as the main sources of information, including specialized databases as CollecTF, RegulomePA, and PDB, among others. The integration of this information allowed us to classify the proteins in terms of 30 functional categories. Finally, an SQL database and a website displaying all the information can be accessed at https://entraf.iimas.unam.mx.

## Supplementary Material

baaf071_Supplemental_Files

## Data Availability

Database homepage: https://entraf.iimas.unam.mx and GitHub repository: https://github.com/BioIIMAS/ENTRAF/. The database is freely available without restrictions for use in academic research.
