# Supplementary figures and images for: ENcyclopedia of TRAnscription Factors in Bacteria and Archaea genomes (ENTRAF) version 2.0
Source: Database (Oxford). 2025 Oct 29;2025:baaf071. doi: 10.1093/database/baaf071 (PMC12569306; doi:10.1093/database/baaf071)

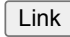

Supplement: baaf071_Supplemental_Files [file baaf071_supplemental_files.zip › Supplementary_material_S1.pdf]

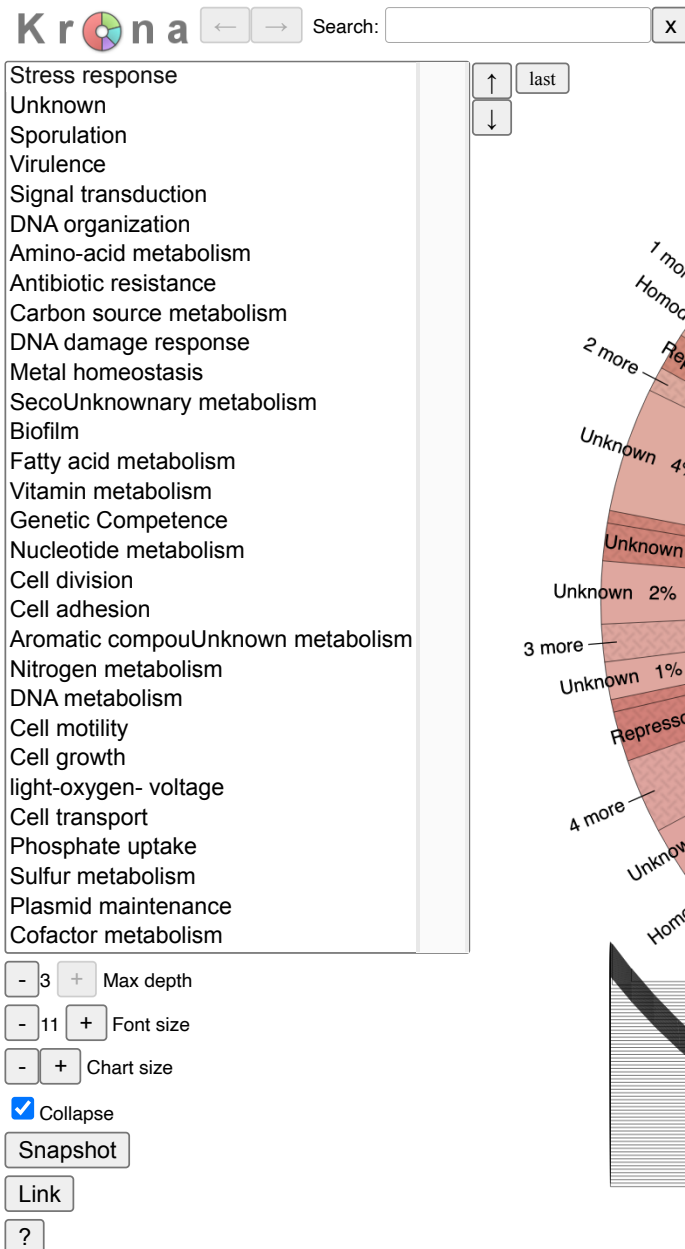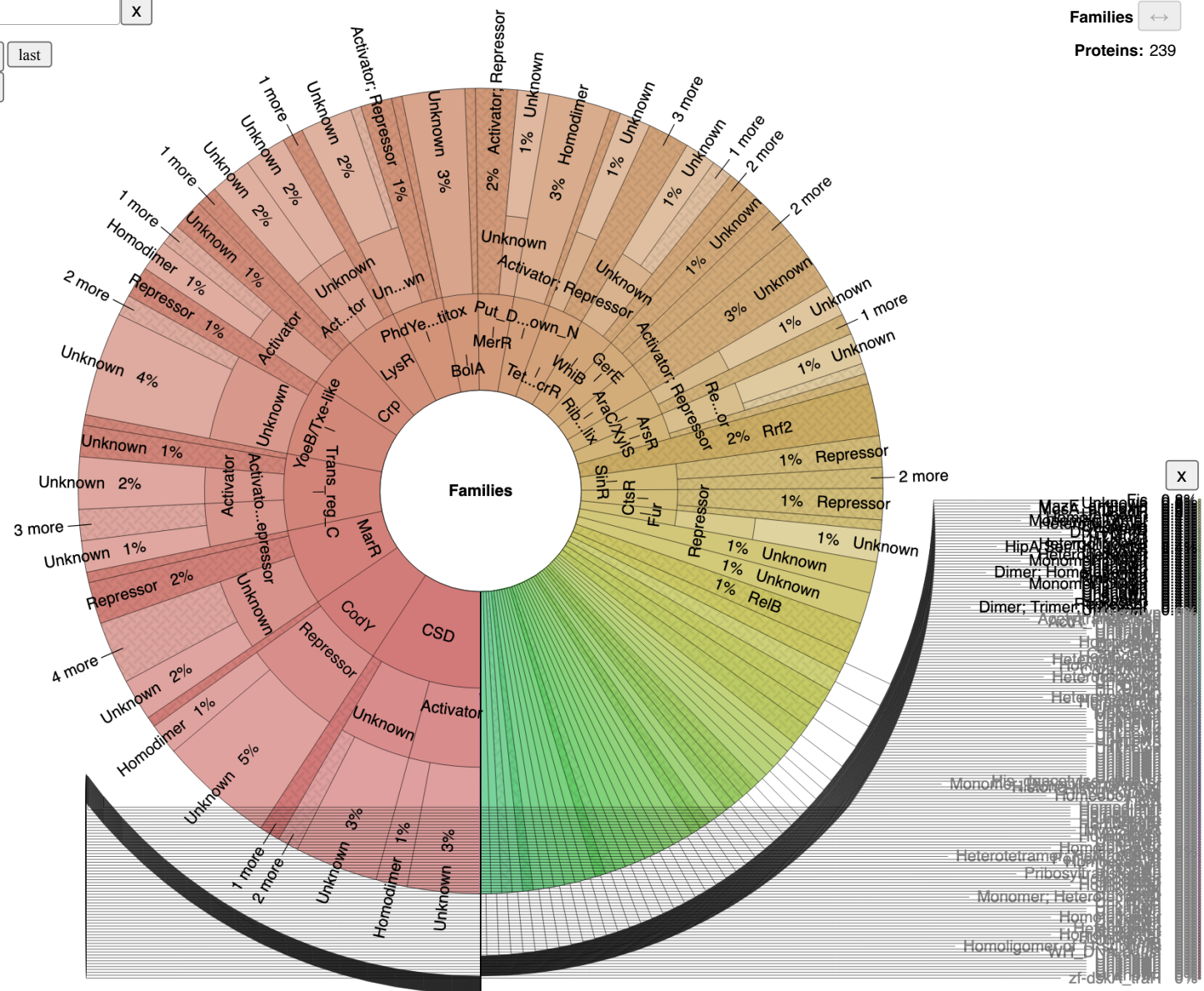

Supplement: baaf071_Supplemental_Files [file baaf071_supplemental_files.zip › Supplementary_material_S3.pdf]
